# Whole-Genome Sequence Data Uncover Widespread Heterothallism in the Largest Group of Lichen-Forming Fungi

**DOI:** 10.1093/gbe/evz027

**Published:** 2019-02-04

**Authors:** David Pizarro, Francesco Dal Grande, Steven Don Leavitt, Paul Stanley Dyer, Imke Schmitt, Ana Crespo, Helge Thorsten Lumbsch, Pradeep Kumar Divakar

**Affiliations:** 1 Departamento de Farmacología, Farmacognosia y Botánica, Facultad de Farmacia, Universidad Complutense de Madrid, Spain; 2 Department of Biological Sciences, Institute of Ecology, Evolution and Diversity, Goethe Universität and Senckenberg Biodiversity and Climate Research Centre (SBiK-F), Frankfurt am Main, Germany; 3 Department of Biology and M.L. Bean Life Science Museum, Brigham Young University, Provo, Utah; 4 School of Life Sciences, University of Nottingham, United Kingdom; 5 Science & Education, The Field Museum, Chicago, Illinois

**Keywords:** lichen-forming fungi, mating system, heterothallism, MAT, sexual reproduction

## Abstract

Fungal reproduction is regulated by the mating-type (*MAT1*) locus, which typically comprises two idiomorphic genes. The presence of one or both allelic variants at the locus determines the reproductive strategy in fungi—homothallism versus heterothallism. It has been hypothesized that self-fertility via homothallism is widespread in lichen-forming fungi. To test this hypothesis, we characterized the *MAT1* locus of 41 genomes of lichen-forming fungi representing a wide range of growth forms and reproductive strategies in the class Lecanoromycetes, the largest group of lichen-forming fungi. Our results show the complete lack of genetic homothallism suggesting that lichens evolved from a heterothallic ancestor. We argue that this may be related to the symbiotic lifestyle of these fungi, and may be a key innovation that has contributed to the accelerated diversification rates in this fungal group.

## Introduction

Sexual reproduction in filamentous fungi is controlled by genes of the mating-type locus (*MAT1*) (Coppin et al. 1997; Kronstad and Staben 1997). This locus comprises two highly dissimilar allelic variants, the *MAT1-1* and *MAT1-2* idiomorphs (Metzenberg and Glass 1990). These variants encode highly divergent proteins: a region encoding an α1 domain characterizes the core *MAT1-1* gene, while a MATA_HMG (high-mobility group)-box characterizes the core *MAT1-2* gene (Turgeon and Yoder 2000). The transcription factors of the MATA_HMG domain are involved in sexual development and have been proposed to be the ancestral fungal sex determinant in fungi (Idnurm et al. 2008; Lee et al. 2008). The molecular function of the α-box is still unclear, although evidence suggests that it may act as transcriptional coactivator (Hagen et al. 1993).

Fungal mating systems can be classified based on the genic content of the *MAT1* locus as, in general, sexual reproduction requires the expression of genes from both *MAT1* idiomorphs (Ni et al. 2011; Dyer et al. 2016). Individuals of heterothallic (out-breeding) species possess genes from only one of the two idiomorphs. Individuals of heterothallic species are thus obligately out-crossing as they require a compatible partner for sexual reproduction to occur (Dyer 2008).

On the other hand, homothallism is an umbrella term that describes a variety of distinct mechanisms that collectively allow for single individuals to be self-fertile and may be classified as primary and secondary homothallism (Wilson et al. 2015). Primary (i.e., genetic) homothallic species possess genes of both *MAT1-1* and *MAT1-2* idiomorphs within a single genome. Secondary homothallism refers to other mechanisms that allow for homothallic behavior such as: 1) uni-/bidirectional mating-type switching when individuals of one or both mating types are able to reversibly (or irreversibly) switch to the opposite mating type forming a mixed, functionally heterothallic colony; and 2) unisexuality when individuals of the same mating type are able to undergo sexual reproduction regardless of the absence of a compatible mating partner. Self-fertility can also be achieved via pseudohomothallism, when opposite mating-type nuclei are packed within a single spore which produces, upon germination, a heterokaryotic, self-fertile mycelium (Whitehouse 1949: Olive 1958; Nelson 1996; Yun et al. 1999; Whittle et al. 2011). All these different systems of secondary homothallism allow, from one side, the preservation of homothallic mating under conditions in which the compatible mating partner is absent or not easily accessible, while retaining the ability to outcross.

Lichen-forming fungi have two alternative reproductive strategies: asexual reproduction and sexual ascospore-producing reproduction. Asexual reproductive systems generally result in the simultaneous propagation of fungal and photosynthetic symbionts (Dal Grande et al. 2012, but see Wornik and Grube 2010), either in granules of varying size containing algal cells and fungal hyphae (soredia or isidia), which are easily detached outgrowths from the lichen thallus. Although asexual reproduction codisperses the fungal and photosynthetic partners, exclusive asexuality has often been interpreted as an evolutionary dead end (Normark et al. 2003). Sexual reproduction decouples the symbionts and the fungus must find a suitable photosynthetic partner for the establishment of a new lichen thallus. While the morphological underpinnings of the reproductive modes in lichens have been dissected in detail (Büdel and Scheidegger 2008), the genetic basis of sexual reproduction in lichens remain, however, largely unknown because of the failure to induce sexuality in vitro (Murtagh et al. 2000).

Primary homothallism is widespread among filamentous ascomycetes, where it is derived from heterothallic ancestors via genetic capture (Beukeboom and Perrin 2014). Self-fertility via homothallism has been proposed to be a prevalent characteristic of lichen-forming fungi (Murtagh et al. 2000), which represent about half of the known ascomycetes (reviewed in Hawksworth 2015).

Primary homothallism has been unequivocally demonstrated for only one lichen-forming fungus of the class Eurotiomycetes, *Endocarpon pusillum*, based on results of whole-genome analysis (Wang et al. 2014). On the other hand, for the Lecanoromycetes, the largest class of lichenized fungi, reports of homothallism have mostly been based on indirect evidence, such as genetic uniformity based on RAPD-PCR fingerprinting of ascospores from the same ascomata (Murtagh et al. 2000; Seymour et al. 2005; Honegger et al. 2007). Obligate, behavioral homothallism has been reported for only two species of the order Teloschistales, *Xanthoria elegans*, and *X. parietina* (Scherrer et al. 2005). In the first case, both mating types were detected in all haploid, single spore isolates, although the exact mating-locus architecture has not been recovered. The latter, instead, represents the firstly reported example of a unisexual lichen species: although genetically heterothallic having lost *MAT1-1*, descendants of meiosis displayed no segregation at the mating locus. Results based on *MAT1* sequencing via PCR amplification and population genetic data have unequivocally demonstrated genetic and behavioral heterothallism for several Lecanoromycetes species from different families representing various reproductive strategies (Ludwig et al. 2017)(Tripp et al. 2017; Dal Grande et al. 2018), such as from predominantly sexually reproducing (e.g., *Parmelina carporrhizans*, Parmeliaceae) (Alors et al. 2017) to predominantly vegetatively reproducing species (*Lobaria pulmonaria*, Lobariaceae) (Singh et al. 2012, 2015). It has been hypothesized that homothallism might be widespread among lichen-forming fungi, especially in the order Lecanorales (Murtagh et al. 2000). It is still unclear, however, whether this reproductive mode represents the ancestral or derived state in lichenized ascomycetes.

In this study, we tested the hypothesis of widespread, ancestral primary homothallism in lichen-forming fungi using genomic data. For this purpose, we gathered whole-genome sequence data and characterized the *MAT1* locus of a set of genomes of lichen-forming fungi representing a wide range of growth forms and reproductive strategies, with particular focus on the Lecanoromycetes, the largest clade of lichenized fungi. Our findings will contribute to the understanding of the regulation of reproductive processes and the evolution of the mating locus in the Lecanoromycetes. This will further contribute to our understanding of the mechanisms behind the accelerated diversification of this important and diverse group of symbiotic fungi.

## Results and Discussion

**Fig. 1.**
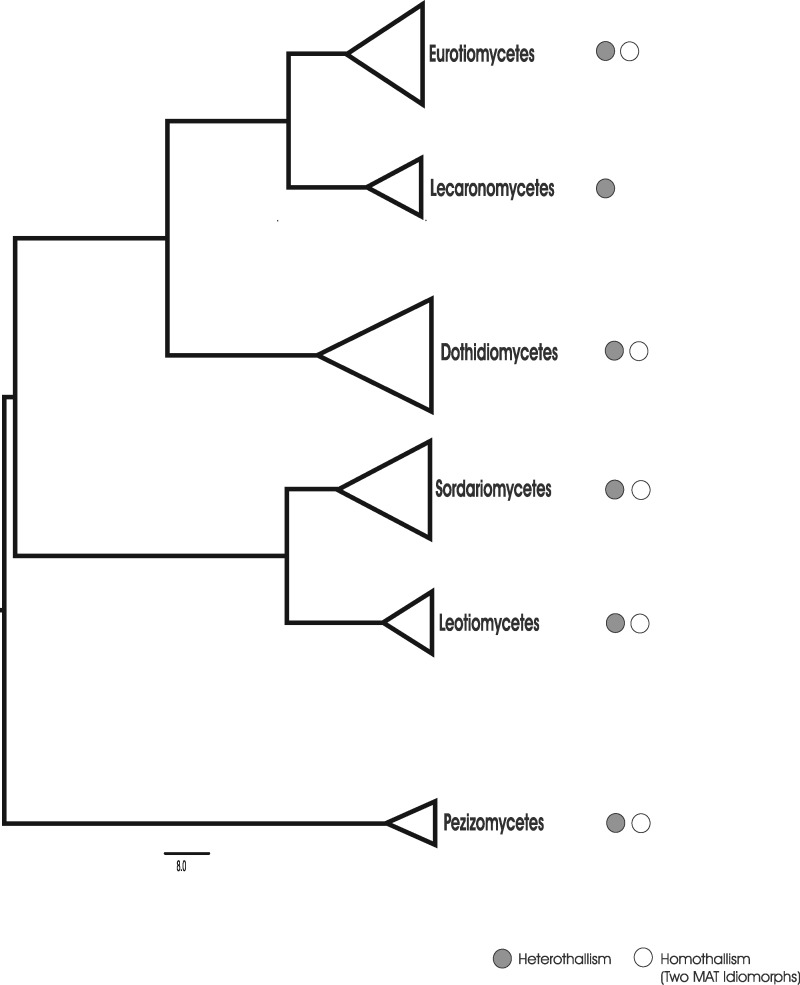
—Phylogenetic placement of the class Lecanoromycetes within the Pezizomycotina. This is a phylogenetic tree from an IQTree analysis based on a concatenated alignment of 81 CEGMA genes. The tree includes 53 species (see supplementary table S2, Supplementary Material online) representing the major groups of Pezizomycotina. The nodes were collapsed at the class rank for clarity of presentation. On the right, gray and white circles represent heterothallic and homothallic organization, respectively. All nodes received maximum ML bootstrap support values (100%).

**Fig. 2.**
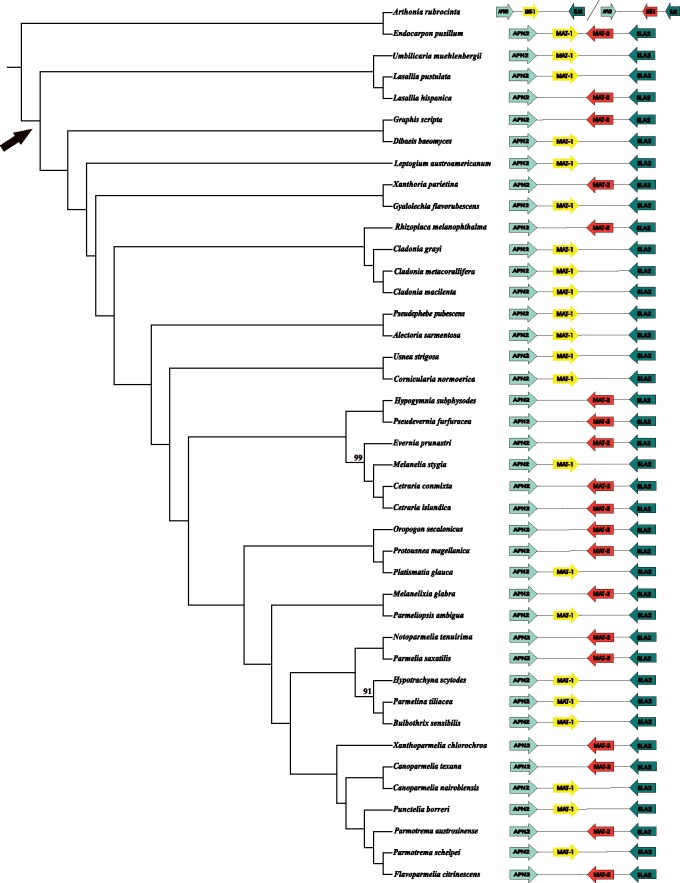
—Left: Evolutionary relationships of 41 lichen-forming fungi based on a concatenated alignment of 735 single-copy protein-coding genes. The tree is a ML tree from an IQTree analysis. Numbers represent ML bootstrap support values based on 1,000 bootstrap pseudoreplicates. Arrow indicates the clade that includes members of the Lecanoromycetes. Right: Schematic representation of the organization of the mating locus in each genome. Except for the two outgroup species that showed homothallic organization either on the same (*Endocarpon pusillum*) or different scaffolds (*Arthonia rubrocinta*), all remaining genomes (i.e., Lecanoromycetes) displayed heterothallic organization.

While a solid foundation on morphological and anatomical understanding of the reproductive modes in lichens have been presented in great detail (Büdel and Scheidegger 2008), the genetic basis of sexual reproduction in lichens remain largely unknown. Here, we analyzed the mating-type locus from 41 genomes representing 4 classes and 9 orders of lichenized fungi. Our results show the loss of primary homothallism in the Lecanoromycetes, the largest group of lichenized fungi (fig. 1). In all 39 Lecanoromycete genomes, we found the same organization of the mating locus, with a single *MAT1* core gene, *MAT1-1* or *MAT1-2*, flanked by the highly conserved cytoskeleton protein (*SLA2*) and DNA lyase (*APN2*) genes (fig. 2 and supplementary table S1, Supplementary Material online). This is in accord with studies reporting heterothallic organization for several species in this group. Sequences flanking the core *MAT1* genes were rather conserved and the transition between similar/dissimilar regions in both idiomorphs was abrupt (supplementary fig. S1, Supplementary Material online). This is similar to the domain organization found in the heterothallic ascomycete *Cochliobolus heterostrophus* (Turgeon et al. 1993). We found a novel gene between *MAT1-1* and *SLA2* in every species containing a *MAT1-1* idiomorph. Within *MAT1-2* loci, a different auxiliary gene was detected between *MAT1-2* genes and *APN2* in every MAT1-2 species, with the exception of *Graphis scripta*. Preliminary phylogenetic analysis suggests that these genes might be lichen-specific (supplementary fig. S2, Supplementary Material online): the auxiliary *MAT1-1* genes of Lecanoromycetes clustered together forming an unsupported monophyletic group with an unsupported sister relationship to the *MAT1-1-4* gene cluster, the latter commonly found in Eurotiomycetes, for example in *Paracoccioides brasiliensis* (Desjardins et al. 2011); similarly, the auxiliary *MAT1-2* genes also formed an unsupported monophyletic group clustering all Lecanoromycete species, except *Xanthoria parietina*, which clustered with *Aspergillus MAT1-2-4*. Sequence analysis showed high divergence among Lecanoromycetes species; in addition, a different number of introns were found depending on the species (supplementary table S3, Supplementary Material online). Interestingly, in some species, for example, *Cetraria islandica*, *Cetraria commixta*, and *Umbilicaria pustulata* (Dal Grande et al. 2018), the *MAT1-2* auxiliary genes displayed a conserved homeodomain leucine-zipper domain (pfam11569, supplementary fig. S1, Supplementary Material online). The characterization of these new genes warrants further study.

Evolutionary transitions between homothallism and heterothallism have commonly occurred in both directions throughout the fungal kingdom (Lin and Heitman 2007). These transitions between inbreeding and outcrossing are likely a response to biological and/or environmental cues that favor one or the other strategy. It has been suggested that homothallism represents the primary reproductive mode of lichen-forming fungi (Murtagh et al. 2000). The key argument in support of this hypothesis was that, based on initial analyses of MAT gene organization in lichens, homothallism was presumed to be widespread. Our findings suggest the opposite, that is, that heterothallism is the prevalent organization of the *MAT1* locus across the wide taxonomic diversity of the Lecanoromycetes under study, including several supposedly asexual lichens. Furthermore, our results indicate a highly conserved organization and synteny of the *MAT1* locus in lichens. By and large, our results strongly support the hypothesis of an ancestral heterothallic state in lichens. This scenario is thus similar to what has been described for the evolution of breeding systems in other ascomycete genera such as *Cochliobolus* (Yun et al. 1999) and, more recently, *Aspergillus* (de Vries et al. 2017; Ojeda-Lopez et al. 2018).

The lack of primary homothallism in the Lecanoromycetes, the most phenotypically diverse class of lichenized fungi, is somewhat surprising. This may well be influenced by the symbiotic lifestyle of these fungi. On the one hand, from the fungal perspective, heterothallism, or obligate outcrossing, can be considered as a high-risk, high-reward strategy. Some portion of the population may, in fact, not be able to find a compatible mating partner, especially for those species with skewed distributions of the *MAT* idiomorphs. On the other hand, the progeny of successful matings will have higher genetic diversity (Otto 2008). Compared with homothallic systems, outbreeding fungi may display accelerated adaptive evolution and more efficient elimination of deleterious mutations, thus they might be more able to avoid Muller’s Ratchet (Roach and Heitman 2014). This is particularly true in environments with more novel factors (Murtagh et al. 2000). It is thus tempting to speculate that the tendency to engage in more prominent outbreeding might be responsible for the accelerated diversification found in this fungal clade (Gaya et al. 2015; J.P. Huang et al., submitted).

From the perspective of the lichen holobiont, sexual reproduction allows for the possibility of reshuffling of the symbionts to generate novel fungus–alga pairs (Dal Grande et al. 2012). As shown recently, these new associations may be key to expanding a lichen’s niche (Rolshausen et al. 2017). On the other hand, the absence of compatible mating partners in the population in case of obligatory outcrossing lichen-forming fungi would comport the risk of being stuck with suboptimal or maladapted photobionts. In this respect, the widespread heterothallism in the Lecanoromycetes would still be advantageous in the presence of mechanisms that would reduce the cost of sex and avoid the problem of mate finding. Results from literature and our own ongoing research seem to support this scenario.

First, there have been several reports of population and seasonal effects on ascospore discharge and germination in this fungal group. In this regard, for example, seasonality was shown to be the regulating factor in *Cladonia furcata* (Jahns et al. 1979) and a few species of the Parmeliaceae (Ruibal, personal communication) Constantino Ruibal, thallus size in *Umbilicaria pustulata* (Hestmark 1992), *Xanthoparmelia cumberlandia* (Pringle et al. 2003), and thallus age in *Parmelia sulcata* (Honegger et al. 2007). This would mean that, like in many other fungi, the timing of sexual reproduction in lichens could be adjusted to when the costs are lowest (Lee et al. 2010; Stelzer 2015). The reproductive strategy being selected for a particular species would therefore depend on interactions among many factors, either environmental (e.g., nutrient availability, competition for space and/or photobiont pools) and/or biological (e.g., population structure, thallus age, and size).

**Fig. 3.**
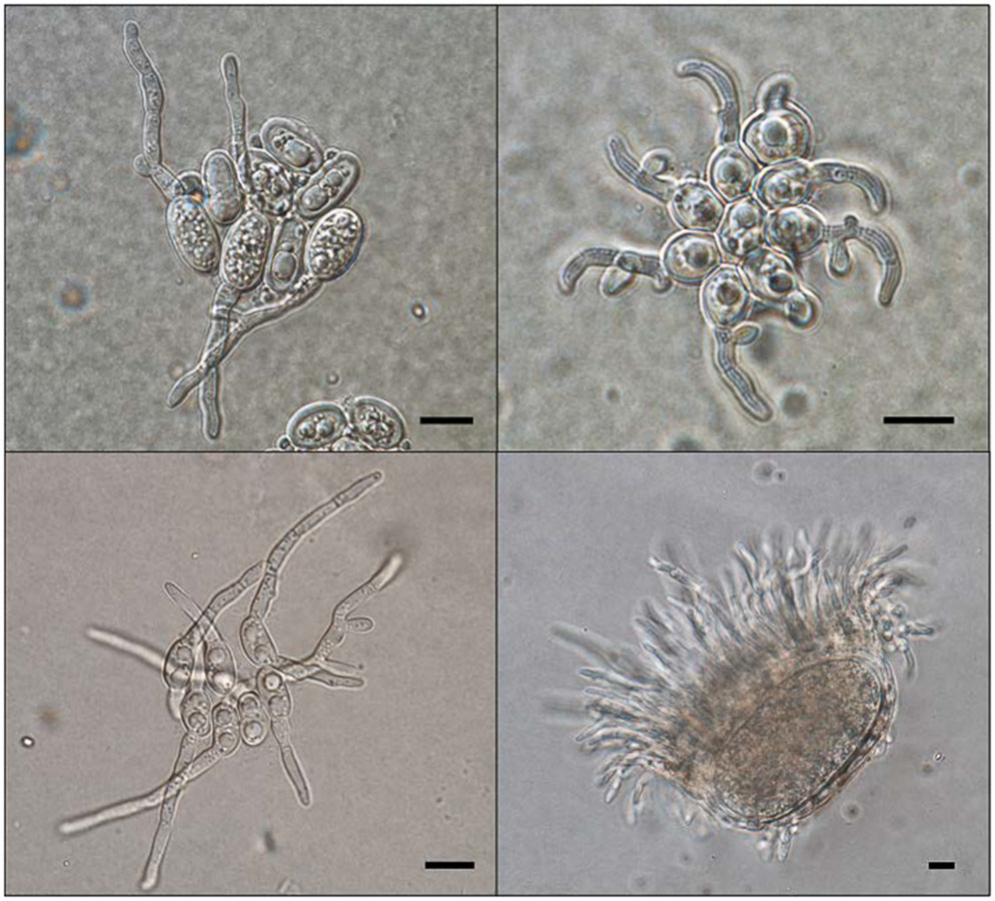
—Top: Simultaneous, unipolar germination of spores ejected from one ascus in *Melanelixia glabra* (Parmeliaceae, Lecanoromycetes; left) and *Cetraria sepincola* (Parmeliaceae, Lecanoromycetes; right) at 9 and 8 days after ejection, respectively. Bottom: bipolar (*Xanthoparmelia stenophylla*, Parmeliaceae, Lecanoromycetes; left) and multipolar (*Menegazzia cincinnata*, Parmeliaceae, Lecanoromycetes; right) spore germination at 7 and 14 days after ejection, respectively. Ascospore isolation and germination followed the method by Molina and Crespo (2000). Scale bars represent 10 μm.

Second, data suggest that many species in this group may be secondarily homothallic. Secondary homothallism in the Lecanoromycetes consists of different strategies or a combination of them, such as unisexuality, the formation of heterokaryotic, self-fertile thalli, and pseudohomothallism. Scherrer et al. (2005) demonstrated unisexuality in the invariably fertile *Xanthoria parietina* as, although being genetically heterothallic, all descendants of meiosis contained only *MAT1-2*. A unisexual cycle may be essential for lineage survival when conditions are unfavorable for heterosexual mating or compatible mating-type partners are not available. This is, for instance, the case for species of the genus *Cryptococcus* that are able to produce spores only via a unisexual or heterosexual cycle (Billiard et al. 2012; Fu et al. 2015). The presence of unisexual mating indicates that in certain ecological niches (e.g., for ruderal species and pioneer colonizers) there may be strong evolutionary pressure for homothallism to arise as the derived state. Unisexual reproduction utilizes a similar genetic pathway as heterosexual reproduction (Feretzaki and Heitman 2013). As such, unisexually derived meiotic spores carry clear advantages over clones or mitotic spores (conidia) in terms of survival rates, especially in adverse environmental conditions (Trapero-Casas and Kaiser 2007). Another advantage of the maintenance of sex via unisexuality may be the reduction of the number of transposons in the genome via increased selection (Roach et al. 2014). The formation of heterokaryotic, self-fertile thalli may be achieved via the joint dispersal and germination of ascospores from the same ascus. The joint ejection and germination of ascospores from *Xanthoria* species, typically early colonizers, was reported by Molina and Crespo (2000) and Honegger et al. (2004). The authors reported that, after only two days from ejection, a mucilage of unknown origins would glue the germinating spores together. We observed a similar phenomenon in several species of the Parmeliaceae (fig. 3, top). This suggests that lichen thalli of the Lecanoromycetes might be often composed of mycelia of opposing mating types, thus being *de facto* self-fertile. This would also mean that lichen thalli would comprise multiple mycobiont genomes, thus rendering metagenomic assemblies further challenging (Meiser et al. 2017; Tripp et al. 2017). The fact that we could retrieve a single, complete mating-type locus in all analyzed metagenomes, could be explained by a skewed mating-type ratio in the thallus portion that was used for DNA extraction. In pseudohomothallism, self-fertility is the result of the packaging of opposite mating-type nuclei within a single spore (Wilson et al. 2015). Although pseudohomothallism in lichen-forming fungi has not yet been reported, we observed bi- or multipolar germination of ascospores in members of the Parmeliaceae (Lecanoromycetes), suggesting the presence of multinucleated spores (fig. 3, bottom) (Molina and Crespo 2000). Multinucleate ascospores are a common feature in the order Pertusariales (Lecanoromycetes) (Pyatt 1968). Future studies should thus focus on characterizing the ascospore mating type in these species.

### Conclusions

This is the first broad scale study dissecting the genetic architecture of the mating locus in lichen-forming fungi. We characterized the *MAT1* locus in the genomes of several lichen-forming fungal species representing a wide range of growth forms and reproductive strategies (isidia, soralia, and ascospores). Noteworthy, we showed widespread heterothallism in the largest, phenotypically most heterogeneous group of lichen-forming fungi. We hypothesize that this is related to the symbiotic lifestyle of this fungal group. Furthermore, the consistency of this character allows us to speculate that this may be implicated in the accelerated diversification rates found in the Lecanoromycetes (J.P. Huang et al., submitted). As such, our study sets the stage for further exploration of the reproductive strategy of lichens, as well as of its evolutionary outcomes.

## Materials and Methods

### Taxon Sampling

A total of 41 lichen-forming fungal species were included in this study (supplementary table S1, Supplementary Material online). We included genomes of species belonging to different classes and orders of lichen-forming fungi. In the class Lecanoromycetes, we analyzed genomes of species belonging to the order Teloschistales (*Xanthoria parietina* and *Gyalolechia flavorubescens*), Umbilicariales (*Umbilicaria pustulata* [Dal Grande et al. 2017; Dal Grande et al. 2018], *Umbilicaria hispanica* [Dal Grande et al. 2018] and *Umbilicaria muehlenbergii*), Ostropales (*Graphis scripta*), Peltigerales (*Leptogium austroamericanun*), and Lecanorales (family Cladoniaceae: *Cladonia grayi*, *C. macilenta*, *C. metacorallifera*; Cladonia macilenta, Cladonia metacorafilera Lecanoraceae: *Rhizoplaca melanophthalma*; Icmadophilaceae: *Dibaeis baeomyces*; Parmeliaceae: 27 species representing six of its seven major clades; see supplementary table S1, Supplementary Material online). Additionally, we included two species belonging to the sister class Eurotiomycetes (*Endocarpon pusillum*) and Arthoniomycetes (*Arthonia rubrocinta*).

### DNA Isolation and Sequencing

Total genomic DNA of 27 specimens of Parmeliaceae were extracted from apothecia or thalli using the Quick-DNA Fungal/Bacterial Miniprep Kit (Zymo Research, Irvine, CA) following the manufacturers’ instructions. DNA concentration was measured using the Qubit dsDNA BR Assay kit (Thermo Fisher Scientific, San Diego, CA). Paired-end libraries (250 bp) were built either using TrueSeq or Nextera XT DNA library preparation kits (Illumina, San Diego, CA). Sequencing of Nextera XT libraries was carried out by the University of Illinois at Chicago Research Resource Center (Chicago, IL) on Illumina NextSeq platform while TruSeq libraries were sequenced on Illumina MiSeq platform at the Pritzker Laboratory for Molecular Systematics and Evolution at The Field Museum, Chicago, IL.

### Trimming, Assembly, and Taxonomic Assignment

Raw sequences were downloaded from an Illumina BaseSpace application and quality-trimmed and filtered using Trimmomatic-0.36 (http://ww.usadellab.org/cm/? page=trimmomatic) Properly working, 18 February 2019 access (Bolger et al. 2014). Bases were trimmed when the average quality of 5-base sliding windows was <20 and bases at the start and end of reads had a quality <3 and 10, respectively. Subsequently, all trimmed reads shorter than 36 bp were filtered out. The same trimming procedure was carried out for genomes retrieved from NCBI, that is, *Arthonia rubrocincta* (PRJNA256244) and *Graphis scripta* (PRJNA256475), and the metagenomes of *Leptogium austroamericanum* (PRJNA256476) and *Dibaeis baeomyces* (PRJNA256246).

Trimmed paired-end reads were assembled using SPAdes or MetaSPAdes (Nurk et al. 2017), depending on the type of data, using default parameters. In order to extract lichen-forming fungal contigs from the respective metagenomic assemblies, scaffolds of each metagenome were subjected to BLASTX (Altschul et al. 1990) searches against a custom database comprising the protein sets of the NCBI nr database (downloaded in August 2016), and additionally, four Parmeliaceae genomes generated from axenic cultures from species of Parmeliaceae (*Cetraria islandica, Parmelina carporrhizans*, unpublished; *Evernia prunastri* and *Pseudevernia furfuracea*; Meiser et al. 2017), 150 complete fungal genomes and 20 algal genomes from JGI using DIAMOND (Buchfink et al. 2015). The results of the DIAMOND search were then used as input in MEGAN6 (Huson et al. 2016) for taxonomic assignment (min-support = 1, min-score = 50, top-hit = 10%, no low complexity ﬁltering). Contigs that were assigned as Parmeliaceae were extracted and used in the subsequent analysis.

### Ortholog Identification and Tree Reconstruction

To infer the phylogenetic placement of the class Lecaronomycetes within the Pezizomycotina, we selected 53 genomes representing the major groups of this subphylum (see supplementary table S2, Supplementary Material online). Orthologs genes were recovered using the CEGMA pipeline (Parra et al. 2007). Every genome was explored using a data set containing 458 proteins of Core Eukaryote Genes. The complete CEGMA genes predicted in each genome were extracted and aligned using MAFFT L-INS-i (Standley 2013). A supermatrix was created by concatenating all alignments using FASconCAT.pl (Kück and Longo 2014). Then, in order to optimize information content and data saturation we used MARE (Misof et al. 2013) with iterative steps of gene exclusion, resulting in an optimal subset of 81 genes. Evolutionary relationships were inferred from this subset using ML analysis as implemented in IQTree v1.5.5 with standard model selection (Nguyen et al. 2015). For each analysis, 1,000 bootstrap replicates were calculated using fast bootstrapping option. The resulting tree was drawn using FigTree v 1.3.1 (Rambaut 2009).

We followed a similar procedure to infer the phylogenetic relationships among 39 lichen-forming fungi belonging to the class Lecanoromycetes. For this purpose, we extended the orthologs gene set to 3,156 single-copy genes of Pezizomycotina as implemented in BUSCO v3 (Simão et al. 2015), resulting in a final matrix of 735 genes (see supplementary table S1, Supplementary Material online). All subsequent analyses were carried out as outlined earlier. The genomes of *Endocarpon pusillum* (Eurotiomycetes) and *Arthonia rubrocinta* (Arthoniomycetes) were used as outgroup.

### Mating-Type Locus Identification

In order to identify the mating-type locus in every genome, we first selected protein sequences of *SLA2* of *Xanthoria polycarpa* (CAI59767.1), *APN2* of *Xanthoria parietina* (CAI59775.1), Alpha-domain of *MAT1-1* of *Xanthoria polycarpa* (CAI59771.1), and HMG-domain of *MAT1-2* of *Dufourea flammea* (CAI59780.2) from Scherrer et al. (2005). These proteins and sequence domains were used as queries in tBLASTn (Altschul et al. 1990) searches against every genome assembly. Scaffolds containing more than one sequence query were retrieved and gene prediction was carried out using MAKER2 (Holt and Yandell 2011). Proteins derived from gene prediction were annotated comparing them with KEGG (Kanehisa et al. 2016) and COG databases (Tatusov 2000). Only scaffolds with complete mating-type loci, that is, containing complete anchoring genes (*SLA2*, *APN2*) were included in further analyses.

### Comparison between Two Different Mating-Type Loci

Two loci of opposite mating type from *Umbilicaria pustulata* (Dal Grande et al. 2017; Dal Grande et al. 2018) were aligned using LASTZ (Harris 2007). Sequence conservation and visualization were carried out using Zpicture (Ovcharenko et al. 2004). Regions with >90% of ECR similarity were retrieved. We further extracted the intergenic regions between mating-type genes and the flanking genes of the two loci using BEDtools (Quinlan and Hall 2010). Intergenic flanking regions were aligned with nucleotide sequence of the opposite mating-type locus using MAFFT.

## Supplementary Material

Supplementary data are available at *Genome Biology and Evolution* online.

## Supplementary Material

Supplementary Data

## Notes

**Data deposition:** All figures and tables generated during the study are included in this article and all data used have been deposited in FigShare: https://figshare.com/s/f6236882992a565c3d3c, last accessed February 18, 2019.
