# Supplementary material for: Whole-Genome Sequence Data Uncover Widespread Heterothallism in the Largest Group of Lichen-Forming Fungi
Source: Genome Biol Evol. 2019 Feb 4;11(3):721–30. doi: 10.1093/gbe/evz027 (PMC6414310; doi:10.1093/gbe/evz027)
Supplement: Supplementary Data [file evz027_supp.zip › Supplementary Materials Legends.docx]

**Supplementary Materials**

**Table S1** List of genomes used in this study and their characteristics: reproductive strategy of the species, presence of homeodomain-containing protein in the mating locus, and taxonomy.

**Table S2** Accession numbers of genomes used in the phylogeny shown in Fig.1 and Uniprot entries of auxiliary mating type genes of the species belonging to different orders of Pezizomycotina.

**Table S3**. List of predicted Auxiliary mating type of Lecanomycetes, their gene length and number of introns.

**Fig. S1** Top: Schematic representation of the organization of the two types of mating alleles in *Lasallia pustulata* (Umbilicariaceae, Lecanoromycetes). Bottom: Whole-locus alignment of the two mating-type alleles using LASTZ. Red blocks indicate highly conserved regions between the loci; coding parts are highlighted in blue.

**Fig. S2**. Phylogenetic analysis of the auxiliary mating type genes of Lecanoromycete species, including some other non-lichenized taxa of Pezizomycotina. In order to identify if they are a novel specific genes on Lecanoromycetes, auxiliary mating type of different orders (Supplementary Table S2) were selected and aligned with all recovered new genes using MAFFT. Relationship was inferred using ML analysis implemented in IQTREE based on MAT1-1 and MAT1-2 genes, with 1,000 bootstrap replicates calculated using fast bootstrapping option. Boot strap support values are indicated above the nodes.
