# Supplementary material for: Whole-Genome Sequence Data Uncover Widespread Heterothallism in the Largest Group of Lichen-Forming Fungi
Source: Genome Biol Evol. 2019 Feb 4;11(3):721–30. doi: 10.1093/gbe/evz027 (PMC6414310; doi:10.1093/gbe/evz027)
Supplement: Supplementary Data [file evz027_supp.zip › Table_S1.pdf]

Species	Mating Type	Auxiliary Gene *MAT1-1*	Auxiliary Gene *MAT1-2*	Reproductive strategy	scaffold1	scaffold2	class	order	family	Reference
Alectoria sarmentosa	MAT1-1	present	-	pred. Vegetative	complete		Lecanoromycetes	Lecanorales	Parmeliaceae	
Arthonia rubrocinta	MAT1-1/MAT1-2	absent	-	pred. Sexual	complete *MAT1-1*	complete *MAT1*	Arthoniomycetes	Arthoniales	Arthoniaceae	McDonald et al. 2013
Bulbotrix sensibilis	MAT1-1	present	-	pred. Sexual	complete		Lecanoromycetes	Lecanorales	Parmeliaceae	
Canoparmelia nairobiensis	MAT1-1	present	-	pred. Sexual	complete		Lecanoromycetes	Lecanorales	Parmeliaceae	
Canoparmelia schelpei	MAT1-1	present	-	pred. Sexual	complete		Lecanoromycetes	Lecanorales	Parmeliaceae	
Canoparmelia texana	MAT1-2	-	present	pred. Vegetative	complete		Lecanoromycetes	Lecanorales	Parmeliaceae	
Cetraria conmixta	MAT1-2	-	present	pred. Sexual	complete		Lecanoromycetes	Lecanorales	Parmeliaceae	
Cetraria islandica	MAT1-2	-	present	pred. Sexual	complete		Lecanoromycetes	Lecanorales	Parmeliaceae	
Cladonia grayi	MAT1-1	present	-	pred. Vegetative	complete		Lecanoromycetes	Lecanorales	Cladoniaceae	Armaleo et al. Unpublished
Cladonia macilenta	MAT1-1	present	-	pred. Vegetative	complete		Lecanoromycetes	Lecanorales	Cladoniaceae	Park et al. 2013
Cladonia metacorallifera	MAT1-1	present	-	pred. Sexual	complete		Lecanoromycetes	Lecanorales	Cladoniaceae	Park et al. 2014A
Cornicularia normoerica	MAT1-1	present	-	pred. Sexual	complete		Lecanoromycetes	Lecanorales	Parmeliaceae	
Dibaeis baeomyces	MAT1-1	present	-	pred. Sexual	complete		Lecanoromycetes	Lecanorales	Icmadophilaceae	McDonald et al. 2013
Endocarpon pusillum	MAT1-1-MAT1-2	-	-	pred. Sexual	complete		Eurotiomycetes	Verrucariales	Verrucariaceae	Wang et al. 2014
Evernia prunastri	MAT1-2	-	present	pred. Vegetative	complete		Lecanoromycetes	Lecanorales	Parmeliaceae	Meiser et al. 2017
Flavoparmelia citrinescens	MAT1-2	-	present	pred. Sexual	complete		Lecanoromycetes	Lecanorales	Parmeliaceae	
Graphis scripta	MAT1-2	-	absent	pred. Sexual	complete		Lecanoromycetes	Ostropales	Graphidaceae	
Gyalolechia flavorubescens	MAT1-1	present	-	pred. Sexual	complete		Lecanoromycetes	Teloschistales	Teloschistaceae	McDonald et al. 2013
Hypogymnia subphysodes	MAT1-2	-	present	pred. Vegetative	complete		Lecanoromycetes	Lecanorales	Parmeliaceae	
Hypotrachyna scytodes	MAT1-1	present	-	pred. Sexual	complete		Lecanoromycetes	Lecanorales	Parmeliaceae	
Lasallia hispanica	MAT1-2	-	present	pred. Sexual	complete		Lecanoromycetes	Umbilicariales	Umbilicariaceae	Dal Grande et al. 2018
Lasallia pustulata	MAT1-1	present	-	pred. Vegetative	complete		Lecanoromycetes	Umbilicariales	Umbilicariaceae	Dal Grande et al. 2017
Leptogium austroamericanun	MAT1-1	present	-	pred. Vegetative	complete		Lecanoromycetes	Peltigerales	Collemataceae	McDonald et al. 2013
Melanelia stygia	MAT1-1	present	-	pred. Sexual	complete		Lecanoromycetes	Lecanorales	Parmeliaceae	
Melanelixia glabra	MAT1-2	-	present	pred. Sexual	complete		Lecanoromycetes	Lecanorales	Parmeliaceae	
Notoparmelia tenuirima	MAT1-2	-	present	pred. Sexual	complete		Lecanoromycetes	Lecanorales	Parmeliaceae	
Oropogon secalonicus	MAT1-2	-	present	pred. Sexual	complete		Lecanoromycetes	Lecanorales	Parmeliaceae	
Parmelia saxatilis	MAT1-2	-	present	pred. Vegetative	complete		Lecanoromycetes	Lecanorales	Parmeliaceae	
Parmelinella wallichiana	MAT1-1	present	-	pred. Vegetative	complete		Lecanoromycetes	Lecanorales	Parmeliaceae	
Parmeliopsis ambigua	MAT1-1	present	-	pred. Vegetative	complete		Lecanoromycetes	Lecanorales	Parmeliaceae	
Parmotrema austrosinense	MAT1-2	-	present	pred. Sexual	complete		Lecanoromycetes	Lecanorales	Parmeliaceae	
Platismatia glauca	MAT1-1	present	-	pred. Vegetative	complete		Lecanoromycetes	Lecanorales	Parmeliaceae	
Protousnea magellanica	MAT1-2	-	present	pred. Sexual	complete		Lecanoromycetes	Lecanorales	Parmeliaceae	
Pseudephebe pubescens	MAT1-1	present	-	pred. Sexual	complete		Lecanoromycetes	Lecanorales	Parmeliaceae	
Pseudevernia furfuracea	MAT1-2	-	present	pred. Vegetative	complete		Lecanoromycetes	Lecanorales	Parmeliaceae	Meiser et al. 2017
Punctelia borreri	MAT1-1	present	-	pred. Vegetative	complete		Lecanoromycetes	Lecanorales	Parmeliaceae	
Rhizoplaca melanophthalma	MAT1-2	-	present	pred. Sexual	complete		Lecanoromycetes	Lecanorales	Lecanoraceae	
Umbilicaria muehlenbergii	MAT1-1	present	-	pred. Sexual	complete		Lecanoromycetes	Umbilicariales	Umbilicariaceae	Park et al. 2014B
Usnea strigosa	MAT1-1	present	-	pred. Sexual	complete		Lecanoromycetes	Lecanorales	Parmeliaceae	
Xanthoparmelia chlorochroa	MAT1-2	-	present	pred. Sexual	complete		Lecanoromycetes	Lecanorales	Parmeliaceae	
Xanthoria parietina	MAT1-2	-	present	pred. Sexual	complete		Lecanoromycetes	Teloschistales	Teloschistaceae	Dyer et al. Unpublished
