# Supplementary material for: Whole-Genome Sequence Data Uncover Widespread Heterothallism in the Largest Group of Lichen-Forming Fungi
Source: Genome Biol Evol. 2019 Feb 4;11(3):721–30. doi: 10.1093/gbe/evz027 (PMC6414310; doi:10.1093/gbe/evz027)
Supplement: Supplementary Data [file evz027_supp.zip › Table_S2.pdf]

SPECIES	Acc. Number
Acephala macrosclerotiorum	PRJNA334316
Alternaria alternata	PRJNA371205
Alternaria brassicicola	PRJNA34523
Ascodesmis nigricans	PRJNA247594
Aspergillus clavatus	PRJNA15664
Aspergillus flavus	PRJNA164607
Aspergillus fumigatus	PRJNA18733
Aspergillus wentii	PRJNA170886
Aspergillus zonatus	PRJNA207688
Aureobasidium pullulans	PRJNA207874
Blastomyces dermatitidis	PRJNA41099
Bulgaria inquinans	PRJNA333781
Chaetomium globosum	PRJNA12795
Chalara longipes	PRJNA213334
Cladonia grayi	JGI/Cgr/DA2myc/ss v2.0
Cladonia macilenta	PRJNA210603
Cladonia metacoralifera	PRJNA219240
Coccomyces strobi	PRJNA340577
Cochliobolus heterostrophus	PRJNA83117
Cochliobolus heterostrophusC5	PRJNA42739
Cochliobolus victoriae	PRJNA160941
Cordyceps militaris	PRJNA41129
Corynespora cassiicola	PRJNA234811
Dothistroma septosporum	PRJNA74753
Endocarpon pusillum	PRJNA181958
Evernia prunastri	NKYR00000000
Fusarium graminearum	PRJNA295638
Fusarium oxysporum	PRJNA347190
Gaeumannomyces graminis	PRJNA37931
Glonium stellatum	PRJNA295956
Grosmannia clavigera	PRJNA39837
Lasallia pustulata	PRJNA275184
Loramyces juncicola	PRJNA334368
Melanospora tiffanyae	PRJNA265602
Meliniomyces bicolor	PRJNA196026
Monascus ruber	PRJNA196033
Mycosphaerella fijiensis	PRJNA337612
Mycosphaerella graminicola	PRJNA19047
Neurospora crassa	PRJNA250607
Neurospora discreta matA	PRJNA207861
Neurospora tetrasperma matA	PRJNA65453
Neurospora tetrasperma mata	PRJNA65453
Parmelina carporhrizans	Unpublished
Pseudevernia furfuracea	NKYQ00000000
Pseudographis elatina	JGI/Pseel1/Pseel1
Pyrenochaeta sp	PRJNA255591
Rhizoplaca melanophthalma	LMCC0506 (GZU)
Spathularia flavida	JGI/Spafl1/Spafl1
Thielavia terrestris	PRJNA32847
Trypethelium eluteriae	JGI/Tryvi1/Tryvi1
Umbilicaria muehlenbergii	JFDN00000000
Uncinocarpus reesii	PRJNA15634
Xanthoria parietina	PRJNA62697

SPECIES	Gene name	Uniprot Entry
Aspergillus lentulus	MAT1-2-4	S5RDM3
Botryotinia fuckeliana	MAT1-2-4	A0A023PIK2
Botrytis_elliptica	MAT1-2-4	A0A0E3TJ69
Ceratocystis fimbriata	MAT1-1-2	X4YZ77
Coccidioides immitis	MAT1-1-4	A7KPA2
Diaporthe sp.	MAT1-1-2	Q1MX51
Diplodia sapinea	MAT1-1-4	V5NSW1
Diplodia sapinea	MAT1-2-5	V5NSE7
Epichloe typhina	MAT1-1-2	Q5TL91
Eutiarosporella pseudodarliae	MAT1-2-5	A0A2D1GT40
Fusarium konzum	MAT1-2-3	G3G2G0
Fusarium mangiferae	MAT1-1-2	G3G2G2
Fusarium mangiferae	MAT1-2-3	G3G2G5
Fusarium xylarioides	MAT1-1-3	B5WWM1
Fusarium_pseudogramineanun	MAT1-1-3	K3VZ10
Gibberella thapsina	MAT1-2-3	G3G2E5
Helicocarpus griseus UAMH5409	AJ79_08761	A0A2B7WQ45
Metarhizium acridum	MAT1-2-3	A0A0P0BZ46
Paracoccidioides brasiliensis	GX48_07139	A0A1E2XVU7
Paracoccidioides brasiliensis	ACO22_06420	A0A1D2J7I2
Paracoccidioides lutzii Pb01	PAAG_05874	C1H533
Paracoccidioides lutzii Pb03	PABG_12218	A0A0A5IEQ3
Phyllosticta citricarpa	MAT1-2-5	A0A1S5QPD1
Pseudogymnoascus destructans	MAT1-1-6	A0A0D3MF52
Pseudogymnoascus destructans	MAT1-2-5	A0A0D3MES0
Pseudogymnoascus roseus	MAT1-1-6	A0A0D3MEY7
Sclerotina_minor	MAT1-1-5	A0A0A0P4J4
Sclerotinia homoeocarpa	MAT1-2-3	H8XXY1
Sclerotinia minor	MAT1-2-4	A0A0A0P2D1
Sclerotinia_sclerotiorum	MAT1-2-4	M1GE12
Trichoderma sp	MAT1-1-2	D2W8Q9
