# Supplementary material for: Whole-Genome Sequence Data Uncover Widespread Heterothallism in the Largest Group of Lichen-Forming Fungi
Source: Genome Biol Evol. 2019 Feb 4;11(3):721–30. doi: 10.1093/gbe/evz027 (PMC6414310; doi:10.1093/gbe/evz027)
Supplement: Supplementary Data [file evz027_supp.zip › Table_S3.pdf]

Species	auxiliary MAT gene	Gene length	Number of introns
Alectoria sarmentosa	Aux_MAT1-1	1075	3
Bulbotrix sensibilis	Aux_MAT1-1	1074	3
Canoparmelia nairobiensis	Aux_MAT1-1	1334	5
Canoparmelia schelpei	Aux_MAT1-1	2974	5
Canoparmelia texana	Aux_MAT1-2	696	2
Cetraria commixta	Aux_MAT1-2	771	2
Cetraria islandica	Aux_MAT1-2	771	2
Cladonia grayi	Aux_MAT1-1	1384	4
Cladonia macilenta	Aux_MAT1-1	1247	5
Cladonia metacorallifera	Aux_MAT1-1	1242	5
Cornicularia normoerica	Aux_MAT1-1	1189	4
Dibaeis baeomyces	Aux_MAT1-1	1050	2
Evernia prunastri	Aux_MAT1-2	1617	2
Flavoparmelia citrinescens	Aux_MAT1-2	2520	2
Gyalolechia flavorubescens	Aux_MAT1-1	1316	4
Hypogymnia subphysodes	Aux_MAT1-2	705	2
Hypotrachyna scytodes	Aux_MAT1-1	781	1
Lasallia hispanica	Aux_MAT1-2	837	2
Lasallia pustulata	Aux_MAT1-1	681	2
Leptogium austroamericanun	Aux_MAT1-1	2322	3
Melanelia stygia	Aux_MAT1-1	1203	3
Melanelixia glabra	Aux_MAT1-2	684	2
Notoparmelia tenuirima	Aux_MAT1-2	576	2
Oropogon secalonicus	Aux_MAT1-2	2213	2
Parmelia saxatilis	Aux_MAT1-2	776	2
Parmelinella wallichiana	Aux_MAT1-1	1073	2
Parmeliopsis ambigua	Aux_MAT1-1	1178	4
Parmotrema austrosinense	Aux_MAT1-2	695	2
Platismatia glauca	Aux_MAT1-1	1130	3
Protousnea magellanica	Aux_MAT1-2	777	2
Pseudephebe pubescens	Aux_MAT1-1	1055	3
Pseudevernia furfuracea	Aux_MAT1-2	1617	2
Punctelia borreri	Aux_MAT1-1	1355	6
Rhizoplaca melanophthalma	Aux_MAT1-2	798	2
Umbilicaria muehlenbergii	Aux_MAT1-1	1255	7
Usnea strigosa	Aux_MAT1-1	1198	4
Xanthoparmelia chlorochroa	Aux_MAT1-2	1625	2
Xanthoria parietina	Aux_MAT1-2	1179	2
