# Supplementary figures and images for: Whole-Genome Sequence Data Uncover Widespread Heterothallism in the Largest Group of Lichen-Forming Fungi
Source: Genome Biol Evol. 2019 Feb 4;11(3):721–30. doi: 10.1093/gbe/evz027 (PMC6414310; doi:10.1093/gbe/evz027)

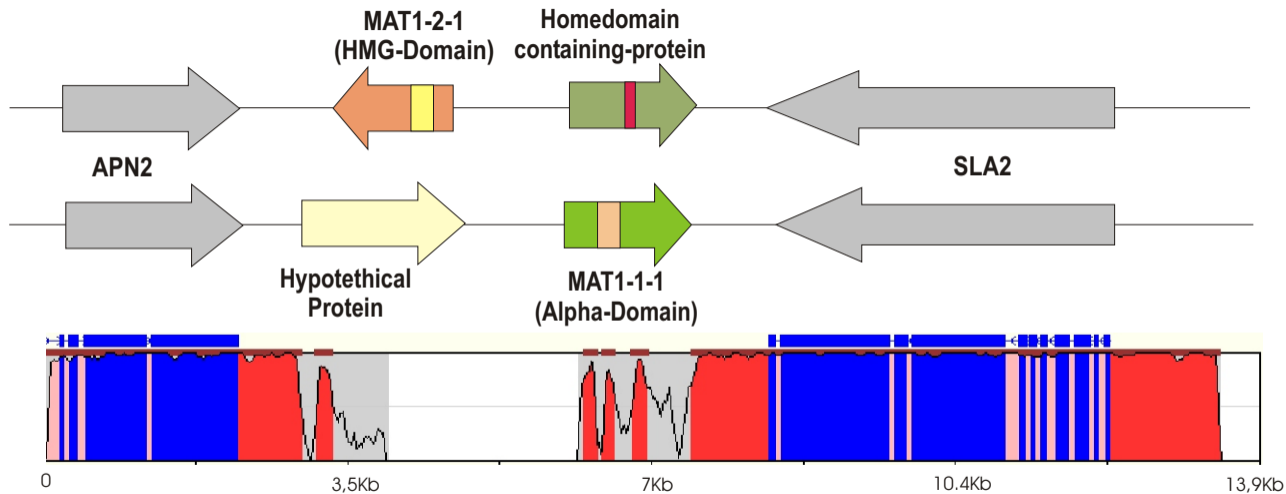

Supplement: Supplementary Data [file evz027_supp.zip › Fig.S1.pdf]

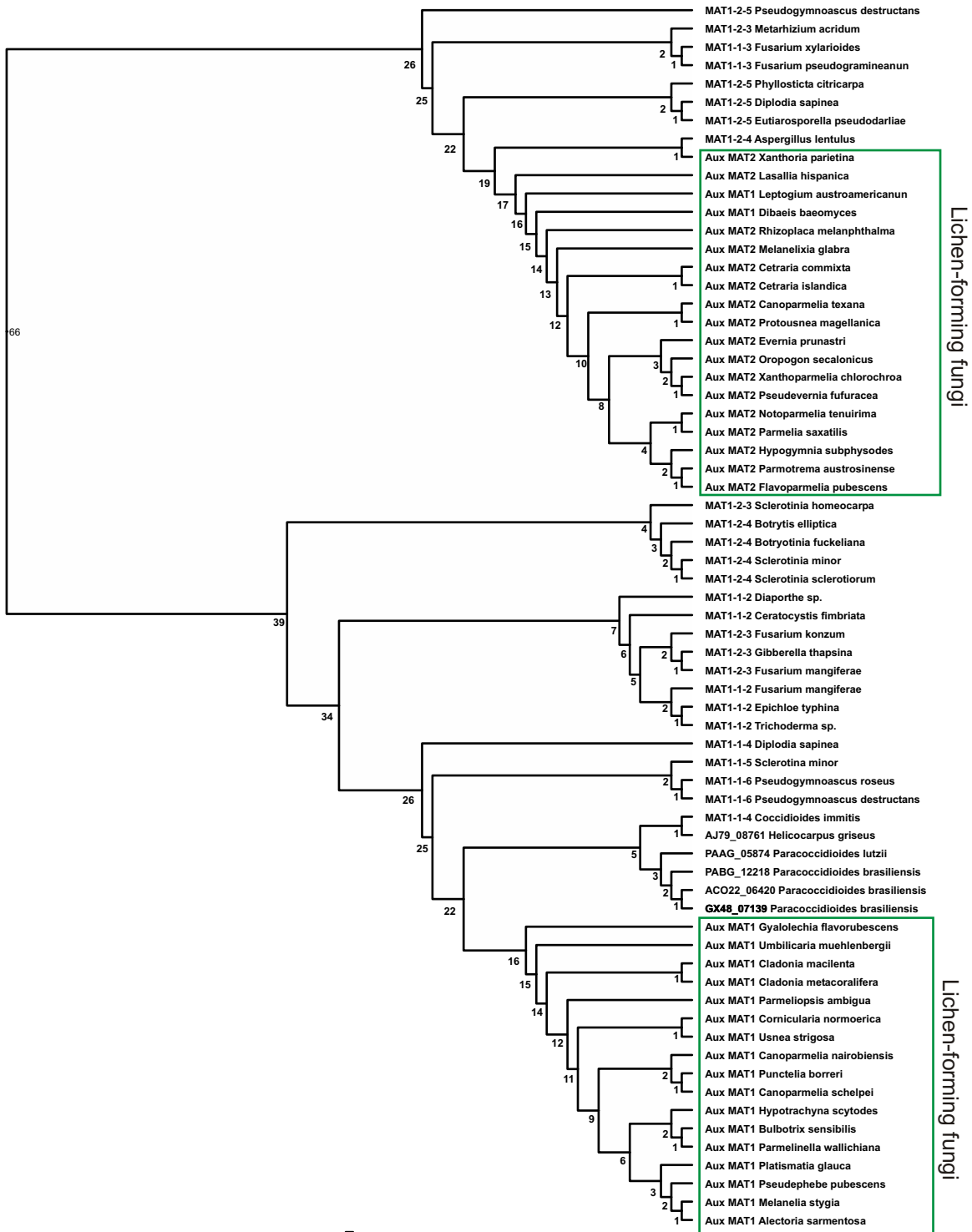

Supplement: Supplementary Data [file evz027_supp.zip › Fig.S2.pdf]
